# Protein Aggregation is Associated with *Acinetobacter baumannii* Desiccation Tolerance

**DOI:** 10.3390/microorganisms8030343

**Published:** 2020-02-28

**Authors:** Xun Wang, Cody G. Cole, Cory D. DuPai, Bryan W. Davies

**Affiliations:** 1 Department of Molecular Biosciences, University of Texas at Austin, Austin, TX 78712, USA; wangxun15@utexas.edu (X.W.); cgcole@uchicago.edu (C.G.C.); 2 Department of Integrative Biology, University of Texas at Austin, Austin, TX 78712, USA; corydubois@utexas.edu; 3 Center for Systems and Synthetic Biology, John Ring LaMontagne Center for Infectious Diseases, Institute for Cellular and Molecular Biology, University of Texas at Austin, Austin, TX 78712, USA

**Keywords:** *Acinetobacter baumannii*, desiccation tolerance, protein aggregation

## Abstract

Desiccation tolerance has been implicated as an important characteristic that potentiates the spread of the bacterial pathogen *Acinetobacter baumannii* on dry surfaces. Here we explore several factors influencing desiccation survival of *A. baumannii*. At the macroscale level, we find that desiccation tolerance is influenced by cell density and growth phase. A transcriptome analysis indicates that desiccation represents a unique state for *A. baumannii* compared to commonly studied growth phases and strongly influences pathways responsible for proteostasis. Remarkably, we find that an increase in total cellular protein aggregates, which is often considered deleterious, correlates positively with the ability of *A. baumannii* to survive desiccation. We show that inducing protein aggregate formation prior to desiccation increases survival and, importantly, that proteins incorporated into cellular aggregates can retain activity. Our results suggest that protein aggregates may promote desiccation tolerance in *A. baumannii* through preserving and protecting proteins from damage during desiccation until rehydration occurs.

## 1. Introduction

*Acinetobacter baumannii* is a hospital-associated pathogen of growing importance and is a paradigm for endemic hospital contamination and epidemic spread [1,2,3,4]. Epidemiological studies combined with genome sequencing of numerous *A. baumannii* clinical isolates have been unable to identify canonical virulence factors, such as toxins, to explain the increase in *A. baumannii*-related infections [5]. The rise of *A. baumannii* as a major hospital-associated pathogen is considered largely due to its ability to quickly acquire antibiotic resistance and to survive on dry surfaces, which could allow spread to new patients through contaminated healthcare equipment and personnel [6,7,8,9,10,11,12]. Several studies have shown that *A. baumannii* can remain viable on dry surfaces for extended periods [8,9,13,14,15]. Despite its apparent importance, our understanding of the molecular details of *A. baumannii* desiccation tolerance is incomplete [1,12,16].

Many factors can influence the ability of an organism to tolerate desiccation, as the removal of water could damage all types of macromolecules by inducing misfolding, aggregation, and oxidation [16,17,18,19,20,21,22]. In *A. baumannii* and other bacteria, several factors have been implicated to increase desiccation survival, possibly by enhancing water retention; these include lipopolysaccharide modifications, capsule formation, and biofilm formation [16,18,23,24,25,26,27,28,29]. In some organisms, desiccation tolerance can be promoted by addition or synthesis of non-reducing sugars, especially trehalose, and induction of hydrophilic stress proteins, both of which can replace water and stabilize macromolecules [18,25,26,27,28,29,30,31]. DNA was often thought to be the critical target for desiccation damage and *A. baumannii* RecA was shown to influence desiccation tolerance [13,14,32,33]. However, another study suggested that bacterial desiccation tolerance correlates with the level of proteome rather than DNA damage [17].

Here we looked into the molecular mechanism of the *A. baumannii* desiccation tolerance. We identified several high-level variables, including cell density and growth phase, affecting *A. baumannii* desiccation tolerance. Interestingly, we identified increased protein aggregation levels as a dominant molecular signal associated with desiccation tolerance. Protein aggregation is commonly viewed as detrimental to protein activity. Remarkably, we found that *A. baumannii* protein aggregates contained functional proteins. Our results suggest protein aggregates may provide a protective repository for proteins during desiccation that can be used to revive *A. baumannii* after rehydration.

## 2. Materials and Methods

### 2.1. Media and Growth Phases

Bacterial strains and plasmids are listed in Table 1. Bacteria were routinely grown in Lysogeny Broth (LB; 10 g tryptone, 5 g yeast extract, 10 g NaCl, in 1 L water) medium at 37 °C with shaking. When required, antibiotics were added to the cultures at the indicated concentrations. Stationary phase bacteria refer to cultures harvested after overnight growth in liquid LB medium, typically at a concentration of A_600_ = 2 (~2 × 10^9^ cfu/mL). Exponential phase bacteria refer to cultures harvested at 3 × 10^8^ cfu/mL, after ~3 h of growth from a 1000-fold-diluted stationary phase culture. Liquid cultures were harvested by collecting 5 mL into a Falcon tube and centrifuge at 2000× *g* for 10 min. Bacteria from LB agar plates refer to colonies grown overnight. LB agar plate samples were harvested by scraping the colonies into 5 mL of water in a Falcon tube. The antibiotics carbenicillin (75 µg/mL), tetracycline (10 µg/mL), and kanamycin (25 µg/mL or 7.5 µg/mL) were added for selection as needed.

### 2.2. Mutant Constructions

We used pSG 25 plasmid for *lacZ* constitutive expression. Since the pSG [36] vectors do not replicate in *A. baumannii*, we constructed a vector pXW1 by adding the pWH1266 origin of replication to pSG25 [37]. pXW2 was generated by PCR amplification of *luc2P* gene to include a myc epitope tag and cloned into pMMB67EH at EcoRI and KpnI. Deletion mutants Δ*abaI* and Δ*abaR* were constructed with recombineering described previously [34].

### 2.3. Desiccation Assay

Desiccation assays were based on methods described preciously [23]. Bacteria from each growth phase were harvested, washed twice with an equal volume of water, and resuspended in water. Samples were adjusted to A_600_ of 1.0, 0.1, and 0.01, and each sample was serially diluted and assayed for input colony forming units (CFU). From these inocula, 10 µL volumes containing 10^5^, 10^6^, or 10^7^ CFU of *A. baumannii* were spotted on 96-well polystyrene plates. The plates were desiccated at 25 °C and at a relative humidity of 40%. After 48 h, 300 μL of water was added into each well to rehydrate the bacteria. The cell suspensions were then serially diluted and plated to determine output CFU. The percent survival was calculated as the ratio of output to input CFU. The differences between the means of each sample group were analyzed by ANOVA. To prepare desiccated samples for aggregates isolation, bacteria were adjusted to a density of 10^6^ CFU/10 µL in 50 mL and desiccated in five 150 mm diameter petri dishes. To speed up the drying process, bacterial suspension was added in droplets of ~100 µL. After 48 h at 25 °C and 40% relative humidity, bacteria were collected and processed.

### 2.4. Water Incubation Tests

Water incubation experiments were performed in the same way as the desiccation assay described above, except that 1 mL of bacterial suspensions at each cell density were kept in Eppendorf tubes for 4 h and then serially diluted and plated to determine survival.

### 2.5. RNA-seq Analysis

We used the *A. baumannii* strain 17978 for RNA-seq analysis to compare its transcriptome in desiccation versus in growth phases. For desiccation, *A. baumannii* grown on an LB agar plate was scraped and diluted in LB medium to A_600_ of 0.5. Bacteria were then spread on polystyrene plates for desiccation. After 24 h of desiccation, duplicate samples were collected for RNA extraction. For the growth samples, *A. baumannii* was grown in liquid LB medium to log phase or stationary phase or on LB agar plates. Samples from each growth phase were collected in duplicate. The cells were pelleted and processed for RNA sequencing as previously described [38]. Sequencing data were mapped to the *A. baumannii* 17978 genome [39], and the reads that mapped to coding sequences (CDS) were analyzed to generate RPKM (Read per Kilobase of transcript, per Million mapped reads) values by CLC Genomics Workbench software. We excluded rRNA and tRNA sequences from analyses.

For principal component analysis, the RPKM values were normalized by the total number of reads mapped to coding sequences of each sample to eliminate batch effect from rRNA and tRNA reads. The 8 samples were scaled and analyzed with PCA using the stats package in R software [40].

Differential expression analyses and annotation tests were performed by CLC Genomics Workbench Software. For the differential expression analyses, RPKM values of the desiccation group were tested against each of the other three growth phases with Baggerley’s test to generate a p-value associated with the weighted proportion fold change [41]. We used an FDR-corrected *p* < 0.01 as the cutoff for a significant change. For annotation test, differentially expressed genes in desiccation versus each of the other three growth phases were annotated using KEGG pathways [42,43,44] and then tested with the Hypergeometric (HyperG) test [45]. The HyperG test generates a p-value associated with the number of genes in each observed pathway in the differential expression analyses compared to the full set of coding sequences. We used *p* < 0.05 as the cutoff for significant over or under-representation of a pathway.

### 2.6. Aggregates Isolation and Quantitation

Cellular protein aggregates were isolated and analyzed as previously described [46]. Briefly, cells were washed and suspended in 360 µL protein buffer B (10 mM potassium phosphate buffer, 1 mM EDTA, pH 6.5). Each sample contains roughly 10^10^ cfu, to which 40 µL of buffer A (Buffer B, 20% *w*/*v* sucrose, 1 mg/mL lysozyme) was added. Cells were lysed by tip sonication at 30% power for 30 s for 3 rounds. After this, samples were centrifuged at 2000× *g* for 15 min to get rid of intact cells in the pellets, followed by a centrifugation of the supernatant at 15,000× *g* to collect insoluble fractions in the pellets. Then the pellets were washed twice with 1% NP40 to wash away membranes proteins. To assay the amount of protein aggregates, samples were normalized by total protein concentration (typically 3 mg/mL), measured by the Bradford assay, and then separated by SDS-PAGE gel and coomassie stained for visualization. Aggregates were quantified with the area density feature in the UVP VisionWorks LS software on an image of the aggregate gel. Samples were assayed in biological triplicates and tested by repeated ANOVA to account for any batch differences caused by gel imaging.

### 2.7. Western Blot

Western blot analysis was carried out by gel transfer to PVDF membranes. The rabbit Anti-beta-galactosidase (Bio-rad, Hercules CA, USA) and rabbit Anti-myc (Upstate) were used at (1:2400) and (1:5000) respectively. The secondary antibody, stabilized peroxidase goat anti-rabbit antibody (Thermo Scientific), was used at 1:1500. Pierce ECL Western Blotting Detection Reagent (Thermo Scientific, Waltham MA, USA) was used for visualization.

### 2.8. ONPG Assay for LacZ Activity

The ONPG assay was performed based on previously described methods [47]. Briefly, 17 µL of cell lysates or purified aggregates was suspended in 253 µL of protein buffer (120 mM KCl, 30 mM HEPES, 5 mM DTT (fresh)) and added to 96-well plates before 30 µL of ONPG solution (5 mg/mL ONPG, 100 mM MgCl_2_ dissolved in the protein buffer) was added for reaction. Reactions were incubated at room temperature for 6 h followed by measuring A_410_. Since the reactions were performed in vitro with no intact cells or cell debri, only the signals at 410 nm were measured. Cell lysates and purified aggregates, each in 3 biological replicates, were normalized by adjusting the total protein concentrations to 3 mg/mL. Aggregates were sonicated by brief treatment with a tip sonicator. Each sample was tested in 4 technological replicates.

### 2.9. Luciferase Assay

Luciferase assays were performed as described [48]. Briefly, *A. baumannii* 17978 wild type strain (WT) and the strain ectopically expressing luciferase (+*luc*) were grown on LB agar plates overnight. The LB plate for the +*luc* strain was supplemented with 0.1 mM IPTG for *luc* expression. Colonies were scraped for protein aggregates purification. A working solution of luciferin was prepared according to the Pierce Firefly Luciferase Glow Assay kit (Thermo Scientific). In a white 96-well plate, aggregate samples were sonicated and diluted in protein buffer (120 mM KCl, 30 mM HEPES, 5 mM DTT (fresh)) before the luciferin working solution was added for reaction. After 60 min of incubation at room temperature, luciferase activity was measured using a GloMax^®^ 96 Microplate Luminometer.

## 3. Results

### 3.1. Cell Density and Growth Phase Influence *A. baumannii* Desiccation Survival

**Figure 1:**
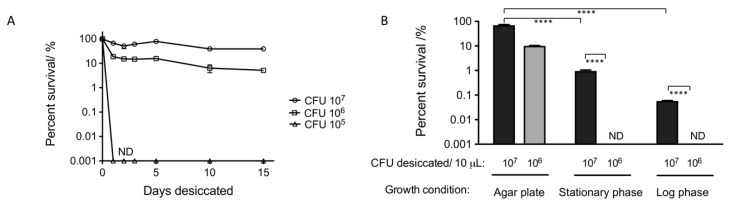
Growth condition and cell density influence desiccation survival of *A. baumannii* strain 17978. (**A**) Percent survival of *A. baumannii* strain 17978 desiccated over 15 days. The strain grown on an LB agar plate was harvested and adjusted to a cell density of 10^7^, 10^6^ or 10^5^ CFU in 10 μL of water to desiccate on polystyrene at 25 °C and 40% relative humidity before rehydration. Cells before desiccation and after 1, 2, 3, 5, 10, or 15 days of desiccation were serially diluted and spotted for CFU to determine the percent survivals. (**B**) Percent survivals of 17978 from desiccation when the strain, prior to desiccation, was grown on LB agar plates or in liquid LB to stationary phase or log phase. Bacteria from all three growth phases were adjusted to a cell density of 10^7^ or 10^6^ in 10 μL of water to desiccate for 48 h before rehydration. Statistical analyses were performed on the log10 transformation of the data. Percent survival comparisons were performed by two-way ANOVA with Bonferroni post-hoc test, and the p-values were presented as the stars in the graphs. **** *p* < 0.0001. ND marks when the percent survival was not detectable. Mean survival with error bars (SEM) was obtained from 3 replicates for (**A**) and 4 replicates for (**B**).

We started out measuring desiccation survival of an *A. baumannii* lab strain ATCC 17978 over 15 days with an assay we developed previously [49]. Colonies grown on LB agar plates were scraped, resuspended in water, and adjusted to three population density groups of 10^5^, 10^6^, and 10^7^ colony forming units (CFU) per 10 µL. Droplets of 10 µL were air-dried to desiccate and then recovered in water to numerate percent survival. In the first 24 h, percent survivals of all three density groups decreased. The survival of the 10^5^ CFU/10 µL density group dropped below the detection limit, whereas survivals of the other two groups remained at around 10~50% (Figure 1A).

By 48 h, the groups of 10^6^ and 10^7^ CFU per 10 µL had reached a relatively stable survival percentage that did not change dramatically throughout the remaining of the 15-day time course. This suggested that *A. baumannii* cells that were capable of surviving desiccation under these conditions had reach their tolerant state by 48 h. Thus, we chose to use the density groups of 10^6^ and 10^7^ CFU per 10 µL at 48 h post desiccation to study additional factors affecting desiccation survival.

As growth phase can impact bacterial response to stress [50,51,52], we tested its effect on desiccation tolerance. Prior to desiccation, *A. baumannii* was grown in rich medium (Lysogeny broth; LB) to exponential or stationary phase or on LB agar plates and processed for desiccation as described above. After 48 h of desiccation, survivals of both exponential and stationary phases were lower than agar plate samples at both input population densities (Figure 1B). *A. baumannii* at each growth phase and population density survived 4 h in water without significant loss of viability (Figure S1), suggesting the loss of viability during desiccation in water is not due to water stress. Our results suggest that initial cell density and growth phase appear to influence desiccation survival.

### 3.2. Transcriptomic Analysis Suggests that Proteostasis is Impacted by Desiccation

Taking an unbiased approach to explore how *A. baumannii* responds to desiccation at the molecular level, we performed a transcriptome analysis on desiccated *A. baumannii* 17978. Bacteria grown on LB agar plates were desiccated for 24 h and RNA was extracted and processed for Illumina sequencing. We needed a comparison condition to assess transcriptional changes under desiccation. However, given the impact growth phase had on desiccation survival (Figure 1B), it was unclear which growth phase comparison would be most appropriate to identify desiccation related transcriptional responses. Therefore, we assessed the *A. baumannii* desiccation transcriptome relative to all three growth phases we had tested, *A. baumannii* grown in liquid LB to exponential or stationary phase or on LB agar plates. To limit the impact of growth medium on our analysis, *A. baumannii* was suspended in LB before desiccation.

**Figure 2:**
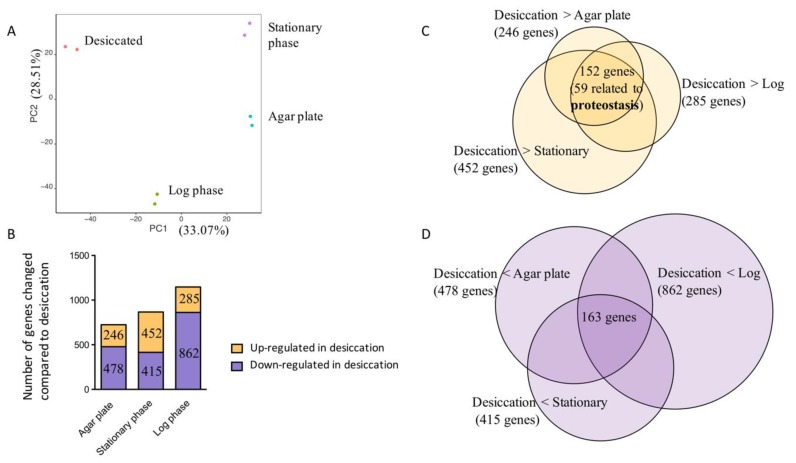
Transcriptomic analysis of desiccated and growing bacteria. (**A**) PCA analysis of the 17978 transcriptomes after desiccation, or after growing in liquid LB to stationary phase or log phase or on an LB agar plate. K-means clustering at *n* = 4 with Pearson distance was performed on the 8 samples. Samples from each condition clustered together as shown by colors of the dots. Desiccated samples and growth samples were all separated. (**B**) The number of genes significantly changed in expression level after desiccation compared to each growth phase at a cutoff of *p* < 0.01. In each comparison, the purple column represents the number of genes down-regulated in desiccation, whereas the yellow one represents the up-regulated genes. (**C**) The overlaps of upregulated genes in desiccation among the three comparisons. (**D**) The overlaps of down-regulated genes in desiccation among the three comparisons.

To determine how variant the transcriptomes were to each other, we performed principle-component analysis (PCA) on the transcriptome data. Figure 2A shows the samples, each presented by a dot, on the plane of the first two principal components (PC1 and PC2), which together account for 61.6% of the variance among the samples. Replicates from each condition clustered together, showing good reproducibility. Samples from different growth phases were scattered on the PC1-PC2 plane, with similar distances to the desiccated samples.

Our results indicate that each growth phase represents a distinct transcriptomic state. Therefore, we performed differential expression analyses of desiccation against each growth phase independently (Figure 2B). There were 246, 452, and 285 genes upregulated in desiccated samples compared to agar plate, stationary phase, and exponential phase samples, respectively (Figure 2B,C). Among these, 152 genes are upregulated during desiccation compared to all three growth phases (Figure 2C). Notably, more than one third of the 152 genes are related to proteostasis, encoding factors for ribosome assembly, chaperone activities, and protein translation and degradation (Table S1). Several genes regulating oxidative stress response were also upregulated, agreeing with previous reports that bacteria experience oxidative stress in desiccation [17,18,19,20,21,22]. In desiccated samples, 478, 415, and 862 genes had lower expression levels compared to agar plate, stationary phase, and exponential phase samples respectively (Figure 2B,D). Among these, 163 genes are shared in all comparisons, most of which related to metabolic functions (Table S1).

Hypergeometric annotation tests on KEGG pathways showed that upregulated genes encoding ribosomal proteins are over-represented in the transcriptome of desiccated samples (Tables S2–S4). These observations suggest that the proteome is likely under stress during desiccation while biosynthesis processes are slowed.

Given the impact of population density on survival (Figure 1A,B), we were curious if quorum sensing genes were differentially regulated under desiccation. Another factor we checked was biofilm production, which has been reported to promote desiccation survival in *A. baumannii* [25]. Neither activities had gene expression changes in all three comparisons. We also tested single gene deletion mutants of quorum sensing (Δ*abaI* and Δ*abaR*) and biofilm production (Δ*pilH* and Δ*pilUT*) for desiccation survival (Figure S2). The genes abaI and abaR were the only pair responsible for quorum sensing in *A. baumannii*. Previous research has shown Δ*pilH* and Δ*pilUT* mutants were defect in biofilm production [34]. None of the mutants showed significantly different survival compared to the wild type strain.

### 3.3. Protein Aggregate Formation Correlates with Desiccation Survival

**Figure 3:**
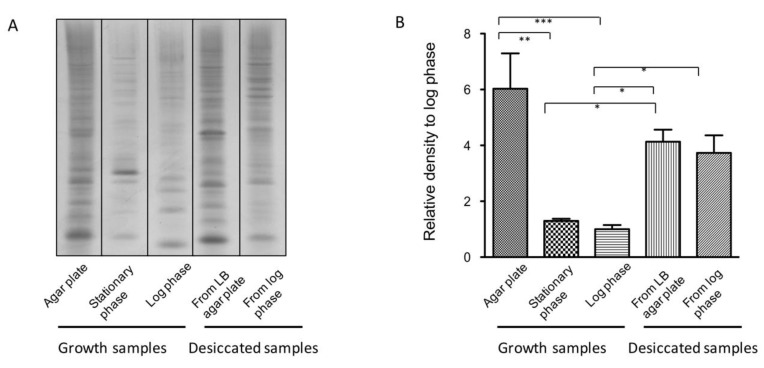
Protein aggregation levels in desiccated and growing bacteria. (**A**) Protein aggregates isolated from 17978 grown on LB agar plates or in liquid LB to stationary phase or log phase, or desiccated. The desiccated samples were grown either on LB agar plates or in liquid LB to log phase prior to desiccation. Aggregates were purified and normalized by total cellular protein concentration [46]. Samples in each panel were processed on the same gel. (**B**) Quantification of (**A**) normalized to the log phase growth sample. Mean area density with error bars (SEM) was obtained from 3 biological replicates. Area density comparisons were performed with Repeated Measures ANOVA followed by Tukey post hoc test. * *p* < 0.05, ** *p* < 0.01, *** *p* < 0.001.

The transcriptomic analyses indicated that the *A. baumannii* proteome is under stress during desiccation and that factors related to proteostasis might play an important role in desiccation survival. Proteome stress can cause proteins to mistranslate, misfold, and/or aggregate [53,54]. We hypothesized that desiccation might cause proteins to misfold, due to a lack of hydrating water, leading to an increase in protein aggregation. To test this hypothesis, protein aggregation levels were assayed in desiccated bacteria and bacteria growing to exponential phase, stationary phase, and on LB agar plates [46]. For desiccated samples, *A. baumannii* 17978 was collected from LB agar plates, suspended in water, and desiccated for 48 h. Protein aggregates were collected, separated by SDS-PAGE, and coomassie stained (Figure 3A).

Based on the transcriptomic analysis, we anticipated aggregation levels to differ between desiccated and growing bacteria. Indeed, the desiccated samples had more protein aggregates than exponential phase or stationary phase samples (Figure 3A,B). Surprisingly, bacteria grown on LB agar plates had a similar level of protein aggregation compared to desiccated bacteria. Since the desiccated samples were initially grown on LB agar plates, it was unclear if the aggregates in desiccated samples were carried over from the LB agar plate growth or if protein aggregates were generated during desiccation. To address this question, we desiccated bacteria growing in exponential phase, which have few aggregates to begin with (Figure 3A). Again, we observed a large amount of protein aggregates after desiccation of a log phase culture, indicating that *A. baumannii* can accumulate aggregated proteins during desiccation. Since bacteria grown on LB agar plates survived desiccation better than log phase or stationary phase samples, it appears that protein aggregation levels pre-desiccation correlate positively with desiccation survival.

### 3.4. Induced Level of Protein Aggregation is Associated with Higher Desiccation Survival

**Figure 4:**
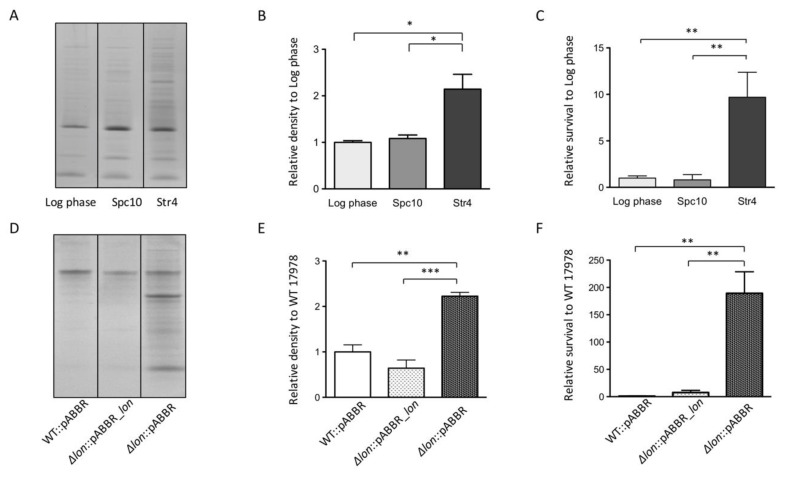
Inducing protein aggregate production is associated with increased desiccation tolerance. (**A**) Protein aggregates isolated from 17978 growing in liquid LB to log phase with no treatment (Log phase), treated with 10 µg/mL spectinomycin (Spc10), or with 4 µg/mL streptomcin (Str4). (**B**) Quantification of (**A**) normalized to Log phase samples. (**C**) Desiccation percent survival of 17978. Bacteria were initially grown in liquid LB to log phase without treatment, treated with 10 µg/mL spectinomycin, or with 4 µg/mL streptomycin prior to desiccation. (**D**) Protein aggregates isolated from the wild type 17978 carrying an empty vector pABBR (WT::pABBR), a *lon* deletion mutant with pABBR (Δ*lon*::pABBR), and a *lon* deletion mutant with *lon* expressed from the vector pABBR (Δ*lon*::pABBR_*lon*). (**E**) Quantification of (**D**) normalized to wild type 17978. (**F**) Desiccation percent survivals of the three strains in (**D**). In B, C, E, F, means with error bars (SEM) were obtained from 3 biological replicates. Comparisons were performed by one-way ANOVA followed by Tukey post-hoc test. * *p* < 0.05, ** *p* < 0.01, *** *p* < 0.001.

The association between protein aggregation level and desiccation survival led us to hypothesize that the aggregates in *A. baumannii* formed while growing on LB agar plates are not detrimental but rather can promote survival. To further test the correlation, we induced protein aggregation in *A. baumannii* growing in exponential phase and assayed desiccation survival. Protein aggregation was induced by sub-minimal inhibitory concentration (MIC) treatment of streptomycin, a ribosome-targeting antibiotic, which increases protein mistranslation and leads to aggregate formation [53]. The *A. baumannii* 17978 in exponential phase was treated with 4 µg/mL streptomycin (below MIC 8 µg/mL) for 30 min and then assayed for desiccation tolerance and protein aggregate levels (Figure 4). A paired culture treated with 10 µg/mL spectinomycin (below MIC 16 µg/mL) served as a control as spectinomycin targets the ribosome without inducing aggregation [53]. Both protein aggregate level and desiccation survival increased after streptomycin treatment (Figure 4A–C). Neither protein aggregate nor desiccation survival changed after spectinomycin treatment.

Similar results were obtained when protein aggregation was induced by deletion of *lon* in 17978. The gene *lon* encodes a well-conserved Lon protease that promotes proteostasis by degrading misfolded proteins and preventing aggregate formation [35,46,55]. As expected, the expression of *lon* was increased in desiccation compared to all three growth phases in our transcriptome analysis (Table S1). We tested how deletion of *lon* impacts aggregate formation and desiccation survival. Both aggregates formation and desiccation survival levels increased in the *lon* deletion mutant compared to the wildtype 17978 (Figure 4D–F). Expression of lon from a plasmid complemented the deletion of *lon* and resulted in similar levels of aggregate formation and desiccation survival compared to the wildtype strain. These results further supported the association between protein aggregate formation and desiccation survival in *A. baumannii*.

### 3.5. Protein Aggregates Contain Functional Proteins

The relationship between protein aggregation and desiccation tolerance suggested that the protein aggregates either directly aid desiccation survival, or they are a reporter of other molecular mechanism(s). In yeast, protein aggregates can preserve functional proteins under stress conditions [54,56]. Similarly, we hypothesize that protein aggregates in *A. baumannii* might preserve some protein function during desiccation.

**Figure 5:**
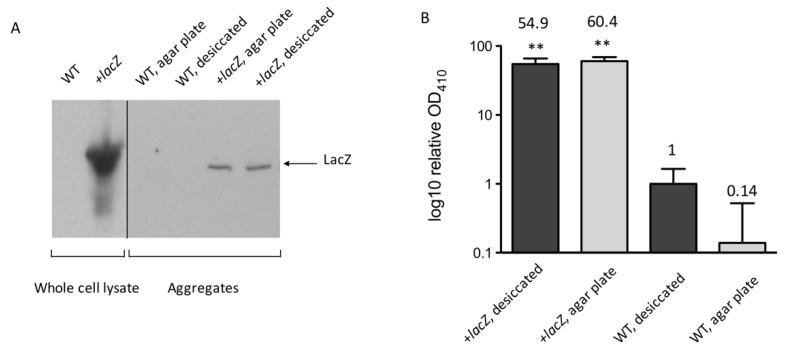
LacZ function detected from *A. baumannii* protein aggregates. (**A**) Western blot for the presence of LacZ. All the samples were prepared from 17978 grown on agar plates. LacZ was detected in the whole cell lysates of 17978 constitutively expressing *lacZ* from the plasmid pXW1 (+*lacZ*). LacZ was also detectable from protein aggregates in the +*lacZ* strain, collected from agar plate growth or after desiccation. LacZ was not detected in whole cell lysates or protein aggregates from the *A. baumannii* parental strain that did not encode *lacZ*. Equal numbers of cells were processed for whole cell lysate samples. Aggregate loading was normalized by total cellular protein concentration. The whole cell lysate samples were processed on the same gel, while the aggregate samples were processed together on a separate gel. (**B**) Protein aggregates were assayed for LacZ activity measuring ONPG cleavage at OD_410_. The signal from buffer was used as baseline and subtracted from all samples. LacZ activity from protein aggregates is reported relative to activity from aggregates isolated from desiccated wild type 17978 (WT, desiccated), which was set at 1. One-way ANOVA with Tukey post-hoc test showed that LacZ activity was higher (** *p* < 0.01) from protein aggregates extracted from *A. baumannii* 17978 ectopically expressing *lacZ*.

To begin to explore this hypothesis, we proceeded to test if protein aggregates isolated from *A. baumannii* grown on LB agar plates or from desiccated samples could contain functional protein. We could not identify an endogenous *A. baumannii* protein that was easily assayed for activity. Therefore, we used LacZ as the reporter protein as its function is readily evaluated. *A. baumannii* does not encode *lacZ* [39]. Accordingly, western blot shows no LacZ protein in wild type *A. baumannii* 17978 (Figure 5A). We transformed *A. baumannii* 17978 with a plasmid pXW1 constitutively expressing *Escherichia coli lacZ* gene (Table 1). LacZ protein was readily detected in this strain by western blot (Figure 5A). Furthermore, *A. baumannii* expressing *lacZ* showed robust β-galactosidase activity compared to buffer and wild type *A. baumannii* alone (Figure S3A).

To test if *A. baumannii* protein aggregates contain functional LacZ, protein aggregates were purified following extensive washing to remove non-aggregated protein and cellular material [46]. As expected, no LacZ signal was detected from aggregates from the parental strain (Figure 5A). LacZ was detectable by western blot in aggregates from all *A. baumannii* samples expressing the *lacZ* gene (Figure 5A). Interestingly, this indicated LacZ was being incorporated into *A. baumannii* protein aggregates. Remarkably, LacZ activity was detected in aggregates from *A. baumannii* expressing the *lacZ* gene growing on LB agar plates and from desiccated samples (Figure 5B). The LacZ activity is unlikely due to LacZ carried over from cell lysate, as the activity remained when we applied more washing cycles on the aggregates before the assay (Figure S3B).

We considered that our results may be an anomaly for LacZ and its relationship with aggregates. We repeated our experiments, using ectopic expression of luciferase (*Luc*). Following the same procedure described for LacZ, Luc protein and activity were also readily detected in *A. baumannii* protein aggregates (Figure S4A,B).

## 4. Discussion

Desiccation is an extreme stress on cells due to the potential for extensive macromolecular damage and depletion of energy caused by dehydration [17,18,19,57,58]. However, as periodic desiccation is common for bacterial niches such as in soil or on human skin, certain bacterial species need to tolerate the wet-dry cycles in order to survive in those environments [18,57]. While sporulation supports desiccation survival of Gram-positive bacteria, it is not clear how Gram-negative bacteria tolerate desiccation without forming spores [1,12,16,59]. In this work, we studied the desiccation tolerance of Gram-negative bacterium *A. baumannii*, as it has been observed to survive desiccation for prolonged periods of time. Since desiccation survival facilitates a number of bacterial pathogens to spread in clinical settings [9], understanding its mechanisms may help lead us to develop better sanitizing strategies to prevent infections.

Many factors influence desiccation tolerance as bacteria likely desiccate in a variety of different environments [17,18,21,22,60]. Therefore, several conditions have been used to study bacterial desiccation tolerance [21,61,62,63,64]. In this work, we chose to desiccate *A. baumannii* at room temperature and humidity in an attempt to mimic settings found in hospitals, and on polystyrene which is commonly used in clinical facilities [65]. The duration of desiccation also influences desiccation survival. For example, the percent survival of *Staphylococcus aureus* from desiccation would first decrease sharply and then flatten out over the time of a week and similarly over a time course of three years [61]. We saw the same trend for *A. baumannii* desiccated in water over two weeks (Figure 1A). Various durations, ranging from minutes to years, have been used for desiccation survival measurement [8,17,21,26,61].

Our study revealed several factors influencing desiccation tolerance under our conditions. Firstly, we observed that the percent survival increased with cell density. A large increase was observed as cell density increased from 10^5^ to 10^6^ cells. Cell density could influence bacterial physiology through several means. By quorum sensing, bacteria could synchronize gene expressions and generate phenotypes at population level [66]. However, quorum sensing deletion mutants showed similar levels of desiccation survival compared to wild type, suggesting it is not a major pathway in desiccation survival under our conditions (Figure 2B). It remains to be tested whether being a source of nutrient, energy, or heterogeneity is the major strength of a large population during desiccation.

Bacteria from different growth phases can display variant phenotypes such as in cell morphology and drug tolerance levels [50,51,52]. We found that *A. baumannii* at each growth phase survive desiccation at different levels. Interestingly, *A. baumannii* survived desiccation better when grown on LB agar plates than when grown in liquid media to either log phase or stationary phase. Since the solid LB agar medium may be more similar to a solid desiccated surface than a liquid medium, bacteria grown on LB agar plates may have a higher percentage of their population pre-adapted for desiccation survival.

Several molecular factors influencing the desiccation survival process have been described previously. For example, oxidative stress has been associated with desiccation [30]. Oxidative stress related genes, such as catalase and oxydoreductase, are turned on during desiccation [67,68]. Antioxidants were found to accumulate in yeast and plants during desiccation, suggesting that dehydrated cells generally experience a higher level of oxidative stress [19,20]. Transcriptome and proteome analyses indicate that desiccation is a complex process involving a wide variety of gene expression and protein production changes [67,68,69,70,71]. In our transcriptomic analysis, genes related to protein translation, folding, and degradation was prominently upregulated in desiccated samples (Figure 2C, Table S1). These include ribosome proteins, chaperones, proteases, and genes related to protein synthesis and ribosome assembly, suggesting that proteome was probably under stress. To limit the impact of growth medium on our RNA-seq analysis, *A. baumannii* was suspended in LB before desiccation, whereas *A. baumannii* was suspended in water for desiccation survival assays. Solutes from LB deposited onto bacteria during the desiccation process could exacerbate osmotic stress. This difference should be considered when extrapolating RNA-seq results to desiccation survival in water alone. Nevertheless, the results of our RNA-seq (proteome stress) were confirmed in subsequent desiccation survival and protein aggregation studies, supporting the quality and value of our RNA-seq analysis to uncover molecular factors affecting *A. baumannii* desiccation survival.

We found an intriguing association between protein aggregation level and desiccation survival in several circumstances (different growth phases, antibiotic treatment, and Δ*lon* mutants) (Figure 3 and Figure 4). The association suggests that protein aggregates either promote desiccation tolerance directly or serve as an indicator of other molecular factor(s). Furthermore, we detected functional proteins from *A. baumannii* aggregates (Figure 5B and Figure S5B). In yeast, research has shown protein aggregation to be reversible and protect proteins from stresses [54,56]. Recent studies in *E. coli* suggested potential roles of protein aggregates in stress responses, although it is still unclear whether aggregates are dissolved or passed down to offspring, or if both could happen depending on the situations [72,73]. Based on our results, protein aggregation might protect some of the *A. baumannii* proteome from damage during desiccation and provide functional proteins for recovery once rehydrated. Further tests would be needed to show the prevalence of this association across *A. baumannii* strains and other bacterial species.

## Figures and Tables

**Table 1:** Bacterial strains and plasmids used in this study.

**Strain**	**Species**	**Genotype/Markers/Infection Pattern**	**Source/Reference**
17978	*Acinetobacter baumannii*	Wildtype lab strain	ATCC
Δ*abaI*	*A. baumannii*	ATCC17978 Δ*abaI*::Kan^r^	This study
Δ*abaR*	*A. baumannii*	ATCC17978 Δ*abaR*:: Kan^r^	This study
Δ*pilUT*	*A. baumannii*	ATCC17978 Δ*pilUT*:: Kan^r^	[34]
Δ*pilH*	*A. baumannii*	ATCC17978 Δ*pilH*:: Kan^r^	[34]
Δ*lon*::pABBR	*A. baumannii*	ATCC17978 Δ*lon*:: Kan^r^ carrying pABBR	[35]
Δ*lon*::pABBR_*lon*	*A. baumannii*	ATCC17978 Δ*lon*:: Kan^r^ complemented	[35]
WT::pABBR	*A. baumannii*	ATCC17978 with empty vector pABBR	[34]
17978 +*lacZ*	*A. baumannii*	ATCC17978 carrying pXW1	This study
17978 +*luc*	*A. baumannii*	ATCC17978 carrying pXW2	This study
**Plasmid**	**Relevant Characteristics**		**Source/Reference**
pABBR	Carb^r^		[34]
pMBB67EH	Carb^r^		ATCC
pSG25	Tet^r^		[36]
pXW1	pSG25 with *A. baumannii* ori for *lacZ* expression; Tet^r^		This study
pXW2	pMMB67EH_*luc2*-myc tag for Luciferase expression; Carb^r^		This study

## Supplementary Materials

The following are available online at https://www.mdpi.com/2076-2607/8/3/343/s1. Figure S1: *A. baumannii* strain 17978 survives incubation in water. Figure S2: Desiccation survivals of quorum sensing and biofilm production deletion mutants. Figure S3: Control groups in the ONPG assay measuring LacZ activity. Figure S4: Luciferase function was detected from *A. baumannii* protein aggregates. Table S1: Genes differentially regulated in desiccation compared to all growth phases. Table S2: Hypergeometric test of differentially expressed genes in desiccated samples compared to agar plate samples. Table S3: Hypergeometric test of differentially expressed genes in desiccated samples compared to stationary phase samples. Table S4: Hypergeometric test of differentially expressed genes in desiccated samples compared to exponential phase samples.
