# Supplementary material for: Protein Aggregation is Associated with Acinetobacter baumannii Desiccation Tolerance
Source: Microorganisms. 2020 Feb 28;8(3):343. doi: 10.3390/microorganisms8030343 (PMC7142981; doi:10.3390/microorganisms8030343)
Supplement: Supplementary file 1 [file microorganisms-08-00343-s001.zip › supplementals/Figure S1-4.docx]

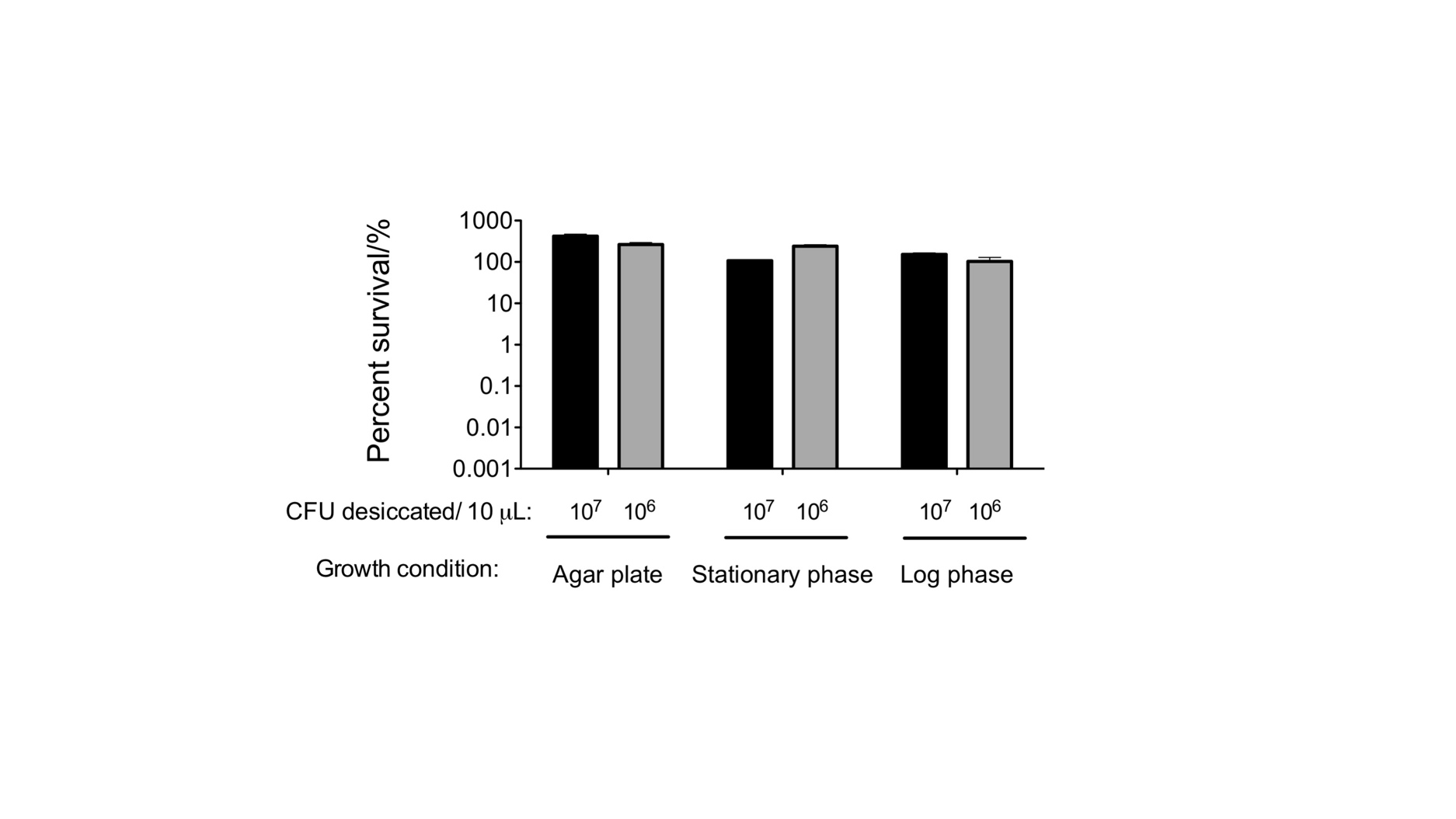


**Figure S1. *A. baumannii* strain 17978 survives incubation in water.** Percent survivals of *A. baumannii* 17978 suspended in water for 4 hours are presented. *A. baumannii* growing on LB agar plate or in LB liquid to stationary phase or log phase were adjusted to a cell density of 10^7, 10^6 or 10^5 CFU per 10 µL in water and 1 mL of each suspension was incubated in Eppendorf tubes at 25 ℃ and 40% relative humidity for 4 hours. Cells before and after incubation were serially diluted and spotted for CFU to determine the percent of bacteria surviving water incubation. Mean survival with *error bars* (SEM) was obtained from 3 replicates.


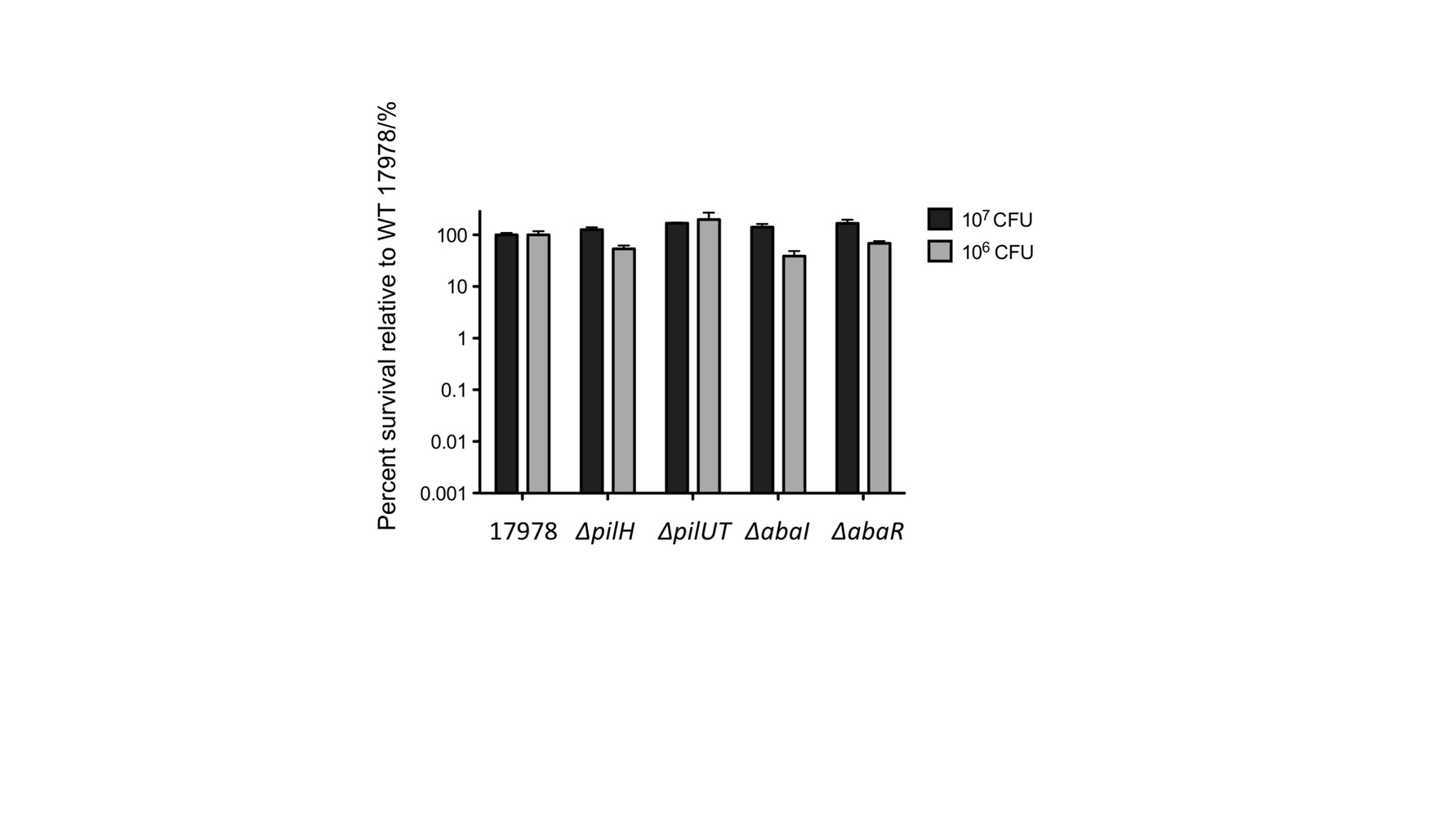


**Figure S2. Desiccation survivals of quorum sensing and biofilm production deletion mutants.** Single mutants of biofilm production (*∆pilH* and *∆pilUT*) and quorum sensing (*∆abaI* and *∆abaR*) were grown on LB agar plates and desiccated for 48 hours before numerated for survival. Percent survivals mutants are normalized to wild type 17978 at each CFU density. Mean survival with error bars (SEM) was obtained from 4 replicates.


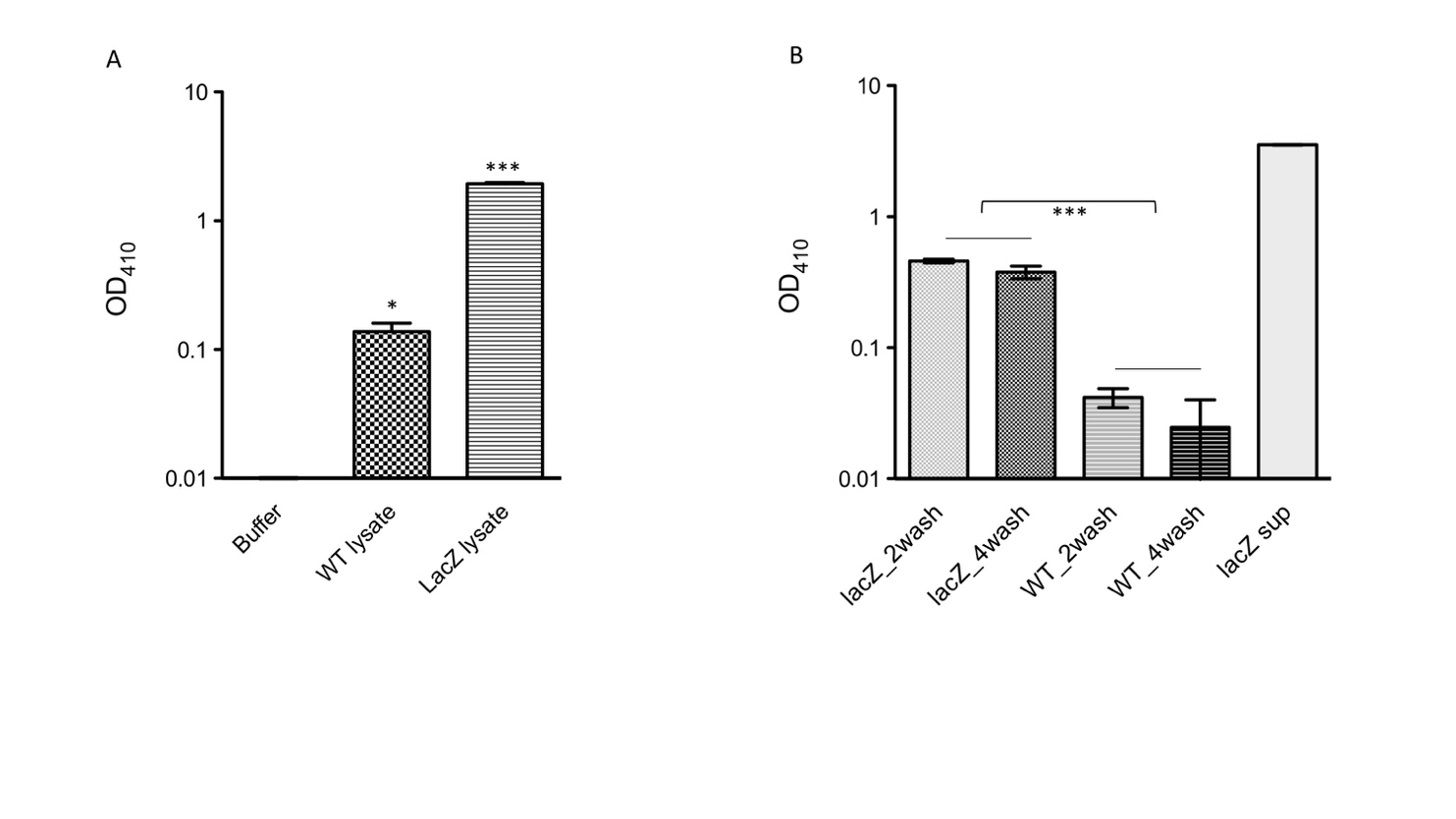


**Figure S3. Control groups in the ONPG assay measuring LacZ activity.** (**A**) The average signals from the buffer was used as baseline and subtracted from all samples. One-way ANOVA with Tukey post-hoc test showed activity from the lysate of *A. baumannnii*17978 ectopically expressing *lacZ (*LacZ lysate) compared to the buffer (* p<0.05, *** p<0.001). (B) LacZ activity of aggregates with 2 washing cycles is similar to that with 4 washing cycles for each sample.


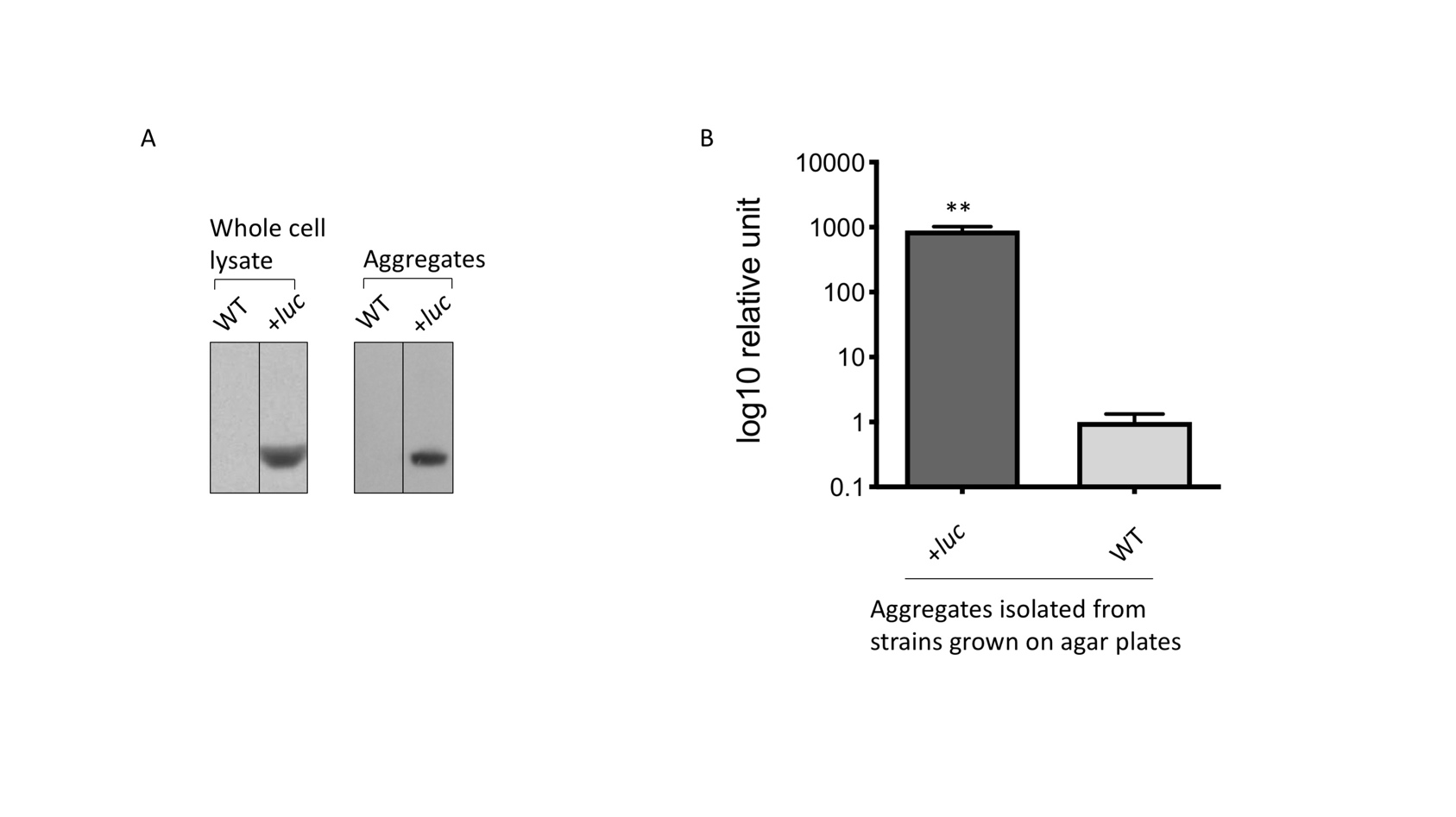


**Figure S4. Luciferase function was detected from *A. baumannii* protein aggregates.** (**A**) Western blot for the presence of Luc. Luc was readily detected from whole cell lysates and protein aggregates of *A. baumannnii* 17978 + pXW2 strain which expressed Luc2 and not detected from the parental strain 17978. Cells were grown on LB agar plates before protein aggregates were extracted. Aggregates loading was normalized by total cellular protein concentration**.**  The samples from whole cell lysates were processed on the same gel and the samples from aggregates were processed on a separate gel. (**B**) Luc assay measuring luciferin oxidation level of aggregates isolated from samples growing on LB agar plates. Buffer-only signal was used as baseline and subtracted from all samples. Values are reported relative to parental *A. baumannii*. One-way ANOVA with Tukey pot-hoc test showed that Luc activity was higher (** p<0.01) from protein aggregates extracted from *A. baumannnii* 17978 ectopically expressing *luc*.
